# Cognitive strategy interventions improve word problem solving and working memory in children with math disabilities

**DOI:** 10.3389/fpsyg.2015.01099

**Published:** 2015-08-04

**Authors:** H. Lee Swanson

**Affiliations:** Educational Psychology, University of California, RiversideRiverside, CA, USA

**Keywords:** math disabilities, strategy training, working memory, cognitive strategies, problem solving

## Abstract

This study investigated the role of strategy instruction and working memory capacity (WMC) on problem solving solution accuracy in children with and without math disabilities (MD). Children in grade 3 (*N* = 204) with and without MD subdivided into high and low WMC were randomly assigned to 1 of 4 conditions: verbal strategies (e.g., underlining question sentence), visual strategies (e.g., correctly placing numbers in diagrams), verbal + visual strategies, and an untreated control. The dependent measures for training were problem solving accuracy and two working memory transfer measures (operation span and visual-spatial span). Three major findings emerged: (1) strategy instruction facilitated solution accuracy but the effects of strategy instruction were moderated by WMC, (2) some strategies yielded higher post-test scores than others, but these findings were qualified as to whether children were at risk for MD, and (3) strategy training on problem solving measures facilitated transfer to working memory measures. The main findings were that children with MD, but high WM spans, were more likely to benefit from strategy conditions on target and transfer measures than children with lower WMC. The results suggest that WMC moderates the influence of cognitive strategies on both the targeted and non-targeted measures.

## Introduction

Although several studies have identified some of the cognitive difficulties in problem solving in children at risk for math difficulties (Swanson and Beebe-Frankenberger, 2004; Andersson, 2010; Fuchs et al., 2010; Geary, 2010), few studies have directly linked deficiencies on cognitive measures to treatment outcomes. One cognitive process that plays a major role in problem solving performance is working memory capacity (WMC). Measures of WMC predict problem solving performance in cross-sectional and longitudinal studies even when measures of calculation, reading, speed, vocabulary, and classroom ratings of inattention have been entered into the regression analyses (Swanson et al., 2008; Zheng et al., 2011). Given the importance of WMC in problem solving performance, this study will test whether strategy instruction compensates for individual differences in WMC in children at risk for math difficulties (MD) on problem solving tasks.

Previous studies show that adjusted post-test scores in problem solving accuracy were a function of the type of strategy instruction implemented as well as WMC capacity at pretest (Swanson et al., 2013b; Swanson, 2014). The interaction was interpreted as suggesting that strategy effects were more pronounced for children with relatively higher WMC than lower WMC. The authors further interpreted their findings as suggesting that children with relatively smaller WMC were overtaxed by certain strategies, which in turn lead to poor learning outcomes (e.g., problem solving accuracy) after training. There were, however, two major problems related to these studies. First, the influence of WMC on problem solving accuracy was *post-hoc* (WMC viewed as a covariate). That is, the authors relied on the pick-point procedure (e.g., Rogosa, 1980) to assess the effects of WMC. Without designating the influence of WMC a priori and as part of the research design, inferences about causality are in question.

The second limitation was that transfer effects to working memory tasks were not directly assessed. Previous studies by these authors (Swanson et al., 2013a; Swanson, 2014) assumed that strategy training would have a positive influence on both problem solving and working memory because both tasks share a common mechanism. This common mechanism was controlled attention specifically, the ability to coordinate process and storage demands despite interfering information (cf. Engle et al., 1999). Unfortunately, their studies did not directly test whether strategy training that directed children's attention to relevant propositions within word problems within the context of interference (i.e., increasing number of irrelevant propositions) would have a positive influence on WM. Although they found transfer to a verbal WM measure (operation span), these findings maybe simply due to training with verbal material rather than directly influencing general WM performance. To address this issue, the concurrent study assesses transfer to both verbal and visual-spatial WM measures.

In summary, the purpose of this intervention study is to determine whether WMC plays an important role in strategy intervention outcomes related to problem solving accuracy in children with MD. Also of interest, is whether strategy instruction that focuses on helping children with MD solve problems, in the context of increasing inference, influences WM performance. In contrast to previous studies that focused on verbal WM (Swanson, 2014), both verbal and visual-spatial WM measures were administered. A randomized control trial was used where children with MD and without MD were assigned to one of three treatment groups: (1) verbal strategies, (2) visual-spatial strategies, or (3) a combination of both verbal and visual-spatial strategies. Embedded within each of the treatment conditions were lesson plans that gradually increased inferring information (the number of irrelevant propositions) within word problems across training sessions. This type of strategy training directed children to attend to relevant propositions while simultaneously increasing irrelevant propositions within the context of the word problem. This training was motivated by several studies showing that learning to differentiate between relevant and irrelevant information is significantly correlated with solution accuracy and students at risk for MD (e.g., Passolunghi and Siegel, 2001; Passolunghi et al., 2001).

To this end, this study addresses three questions:

1). Do cognitive strategies place different demands on WMC in children with MD?

One hypothesis tested is that children with MD who meet a certain threshold of WMC would have spare working memory resources to benefit from cognitive strategies. Because information has to pass through working memory before it can be consolidated into long-term memory, the limited capacity of working memory can be considered the bottleneck for learning. Thus, individuals with MD but relatively higher WMC are better able to utilize cognitive strategies than children with lower WMC. A contrasting hypothesis is that cognitive strategies compensate for the excessive processing demands placed on WMC due to the extraneous load of the problem solving task. Children with relatively low WMC may be more responsive to cognitive strategies because it helps them compensate for working memory limitations. In contrast, children with relatively higher levels of WMC may experience a level of redundancy or unnecessary processing related to strategy training that does not facilitate learning. Thus, we predict that WMC will interact with treatment outcomes (see Swanson, 2014, for further discussion of these hypotheses).

2). Are some cognitive strategies more effective than others for children with MD?

Although several strategy conditions may improve solution accuracy, relative to the control condition, some strategies may play a more important role for children with MD than their average-achieving peers. Previous studies have shown that because the combined strategy draws upon separate verbal and visual-spatial storage capacities, the combination of these storage systems opens up the possibility for more information to be processed (e.g., Mayer, 2005). Thus, the study explores whether a combination of both verbal and visual-spatial strategies may be more beneficial for enhancing problem solving accuracy relative to strategy conditions that emphasize verbal or visual-spatial strategies in isolation.

3). Does practice solving problems that gradually increase irrelevant information influence WM performance?

We assumed that training that includes gradual increases in competing information within the context of relevant information may improve working memory. As previously stated, we do not expect strategy instructions to directly modify WM *per se*, but rather to increase the retrievability of information. Previous studies have attempted to influence WM by teaching WM direct, but these studies have not found changes that extend beyond trained tasks, and therefore have not yielded changes in academic performance (e.g., Melby-Lervåg and Hulme, 2013). Some studies have found a generalization to non-targeted related processes (visual WM training was related to recognizing visual spatial patterns, Klingberg et al., 2005), or a delayed sleeper effect (Holmes et al., 2009) on math, but strategies to improve or compensate for WM limitations has not been shown, at this point, to make direct or substantial improvement on important classroom tasks such as math problem solving performance. Perhaps one of the reasons for the poor transfer is that the WM training has **not** been embedded within academic instruction. Thus, treatment conditions in this study will include training related to identifying irrelevant propositions (sentence) across lesson plans. We assumed that training that includes gradual increases in competing information within the context of relevant information may improve controlled attention, and therefore have influence on working memory performance. Thus, we tested whether WM performance improved as a function of strategy conditions.

## Methods

### Participants

Participants were comprised of 204 third grade students from two public school districts in southern California. The research was carried in accordance of the Human Subjects committee and written informed consent at the University of California-Riverside protocol number (HS-O6-099) and Federal grant number USDE R324A090002 Institute of Education Sciences. Written informed consent was received from parents and/or guardians prior to testing and intervention in accordance with the Declaration of Helsinki. This data was gathered in 2010 as part of a larger research project that occurred from 2009 to 2014. The overall goal of the project was to identify an array of strategy conditions that facilitate problem solving in children with math disabilities. Of the 204 children selected for this study, 101 were female and 103 were male. Ethnic representation of the sample was 116 Anglo, 38 Hispanic, 16 African American, 11 Asian, and 28 mixed and/or other (e.g., Anglo and Hispanic, Native American). The mean SES of the sample was primarily low SES to middle SES based on free lunch participation, parent education, and occupation. However, the sample varied from low middle class to upper middle class.

#### Definition of risk for math disabilities (MD)

The 25th percentile cut-off score on standardized math measures has been commonly used to identify children at risk (e.g., Fletcher et al., 1989; Siegel and Ryan, 1989). Because the focus of this study was on children's word-problem solving difficulties, we examined children who performed in the lower 25th percentile on norm-referenced word-problem solving math tests. We chose to focus on children with MD in grade 3 because this is when word problems are introduced into the curriculum. Our criteria for defining MD was a score between the 25th and 90th percentile on a measure of fluid intelligence (Raven Colored Progressive Matrices Test-RCMT), and a score below the 25th percentile (below a standard score of 90 or scale score of 8) on standardized word problem solving math tests. The story problem subtests from the Test of Math Ability (TOMA, Brown et al., 1994) and Key Math (Connolly, 1998) were used to identify children below the 25th percentile (scale score of 8). This procedure separated the sample into 94 children with MD (46 females) and 110 children (55 females) without MD. Table 1 shows the means and standard deviations for children with and without MD.

**Table 1:** **Comparison of children with and without math disabilities on pretest and moderator variables as a function of high and low WM**.

	**Total sample**			**Math disabled**				**Average achievers**			
	**N =204**			**Low-WM**		**High-WM**		**Low-WM**		**High-WM**	
	**Mean**	**SD**	**Reliability**	**Mean (N = 63)**	**SD**	**Mean (N = 32)**	**SD**	**Mean (N = 45)**	**SD**	**Mean (N = 65)**	**SD**
Age	106.26	7.08		107.17	8.72	108.53	6.36	103.79	5.41	105.92	5.94
**CLASSIFICATION**											
TOMA-S	8.16	2.35	0.84	6.27	1.08	6.84	1.07	9.09	2.20	9.97	2.05
Key-math-S	9.20	3.43	0.93	5.76	2.66	7.33	1.15	11.39	1.98	10.87	2.19
Average-S	9.00	2.99	0.87	6.28	1.91	6.7	1.61	11.57	1.66	11.12	1.59
**FLUID INTELL**.											
Raven-ST	104.46	12.5	0.99	99.35	12.66	100.84	10.92	110	11.14	107.16	11.76
**READING**											
TORC-S	10.47	2.19	0.80	9.48	2.19	9.81	2.17	10.93	1.97	11.42	1.86
WRAT-ST	105.28	12.29	0.81	98.21	10.39	105.56	12.39	106.51	8.72	111.42	12.8
**CALCULATION**											
WIAT_ST	99.36	11.24	0.86	95.16	10.64	98.16	10.65	100.87	9.85	103.27	11.7
WRAT_ST	100.19	11.17	0.81	94.63	10.66	97.91	11.29	103.19	9.22	104.94	10.23
**WORKING MEMORY**											
Rconceptwm-R	5.50	4.78	0.80	3.03	2.13	7.75	4.10	3.44	2.82	8.32	5.98
Rdigsent-R	7.00	4.94	0.84	4.56	2.97	8.75	5.09	5.00	3.15	10.05	5.55
Update-R	6.46	4.46	0.84	3.78	2.58	9.00	4.69	4.19	2.67	9.57	4.25
WM span^a^	−0.04	2.04		−1.64	0.86	1.34	1.28	−1.36	0.88	1.87	1.7
**CRITERION MEASURES**											
CMAT_R	8.06	3.07	0.90	6.14	3.04	7.78	2.80	8.6	2.42	9.75	2.54
Visual matrix-R	13.73	8.51	0.90	11.39	7.20	17.02	10.64	13.31	8.39	14.78	8.09
Oper Span-R	4.67	4.29	0.87	4.23	3.59	4.47	4.25	4.48	4.09	5.37	5.04

_R at the end refers to Raw Score, _S at the end refers to Standard or Scale Score; TOMA, Test of Math Ability; CMAT, Comprehensive Test of Math Abilities; KEY-Math, Key Math test; Average_S, mean scale-score (TOMA, KEYM); WRAT, Wide Range Achievement Test; TORC, Test of Reading Comprehension; CONCEP, conceptual span; Composite

a *= mean z-score of WM span (conceptual span, digit/sentence span, and updating)*.

As shown in Table 1, performance on standardized measures of word problem solving accuracy for the MD sample was below the 25th percentile (scale score at or below 8, standard score below 90), whereas their norm-referenced scores on calculation, reading comprehension and fluid intelligence were above the 25th percentile. No significant differences emerged between children with and without MD as a function of ethnicity, χ^2^_(5, *N* = 204)_ = 1.26, *p* > 0.05 or gender, χ^2^_(1, *N* = 204)_ = 0.005, *p* > 0.10.

### Design and treatment conditions

#### Random assignment

Twenty-two classrooms were randomly assigned to each treatment. All children within each classroom were sent parent permission forms. From the sample of children within each classroom in which permission was granted, a battery of tests were administered to determine children were at risk for MD. Based on the administered tests discussed below, children were stratified as at risk if they performed above or below a median score in WMC based on preliminary data collected in 2009. An approximately equal number of children without MD were randomly selected (stratified by WMC, gender and ethnicity). Thus, the sample included children assigned to a control group (*N* = 56), or to one of three treatment conditions [Verbal-emphasis (*N* = 49), Verbal + Visual Strategies (Diagramming; *N* = 53), and Visual-emphasis (Diagramming; *N* = 46)].

#### Common instructional conditions

All children in the study participated with their peers in their home rooms on tasks and activities related to the district wide math school curriculum. The school wide instruction across conditions was the enVisionMATH Learning Curriculum (Pearson Publishers, 2009). A number of the elements within the curriculum were also utilized in our treatments (e.g., find the pattern, etc…). However, in contrast to the district instruction, our treatment conditions directly focused on specific components of problem solving over consecutive sessions presented in a predetermined order. In addition, the lesson plans for the experimental condition focused directly on the propositional structure of word problems.

#### Experimental conditions

Each experimental treatment condition included 20 scripted lessons administered over 8 weeks. Iterations of the treatment lesson plan are reported in Swanson et al. (2013a; Appendix A in Supplementary Materials). We briefly summarize the procedures here (also see Swanson, 2014, for a complete description).

Each lesson was 30 min in duration and was administered three times a week in small groups of four to five children. Lesson administration was done by one of six tutors (doctoral students). Children were presented with individual booklets at the beginning of the lesson, and all responses were recorded in the booklet. Each lesson within the booklet consisted of four phases: warm-up, instruction, guided practice, and independent practice.

The warm-up phase included two parts: calculation of problems that required participants to provide the missing numbers (9+2 = x, x +1 = 6; x −5 = 1), and a set of puzzles based on problems using geometric shapes. This activity took approximately 3–5 min to complete.

The instruction phase lasted approximately 5 min. At the beginning of each lesson, the strategies and/or rule cards were either read to the children (e.g., to find the whole, you need to add the parts) or reviewed. Depending on the treatment condition, children were taught the instructional intervention (Verbal strategy, Diagramming, or Verbal strategy + Diagramming). The steps for the Verbal-emphasis approach included: find the question and underline it, circle the numbers, put a square around the key word, cross out information not needed, decide on what needs to be done (add/subtract/or both), and solve it. For the Visual-emphasis condition (diagramming) students were taught how to use two types of diagrams. The first one represented how parts made-up a whole. The second type of diagram represented how quantities are compared. The diagram consisted of two empty boxes, one bigger and the other smaller, in which the students were to fill in the correct numbers representing the quantities. An equation with a question mark was presented. The question mark acted as a placeholder for the missing number provided in the box. Finally, for the combined Verbal + Visual (diagramming) Strategy condition, an additional step (diagramming) was added to the 6 Verbal Strategy steps described above. This step included directing students to fill in the diagram with given numbers and identifying the missing numbers (question) in the corresponding slots in the boxes.

The third phase, *guided practice*, lasted 10 min and involved students working on three practice problems. Tutor feedback was provided on the application of steps and strategies to each of these three problems. In this phase, students also reviewed example problems from the instructional phase. The tutor assisted students with finding the correct operation, identifying the key words, and providing corrective feedback on the solution.

The fourth phase, *independent practice*, lasted 10 min and required students to independently answer another set of three word problems without feedback. If the student finished the independent practice tasks before the 10 min were over, they were presented with a puzzle to complete. Student responses were recorded for each session to assess the application of the intervention and problem solving accuracy. In order to make application comparisons across treatment, point values were converted to z-scores. For the Visual–emphasis condition, points were recorded for correctly choosing the correct diagram, correctly filling in the numbers for the diagram, identifying the correct operations, and correctly solving the problem. For the Verbal + Visual-Strategy condition, points were recorded for correctly choosing the diagram, inserting correct numbers, applying strategies, identifying the correct operations, and correctly solving the problem. For the Verbal-emphasis condition, points were recorded for identifying the correct numbers, applying strategies (e.g., underlining), identifying the correct operations, and solution accuracy.

#### Increments of irrelevant propositions

Word problems for each *independent practice* session included three parts: question sentences, number sentences, and irrelevant sentences. For each problem in the independent practice session, at least two number sentences were relevant to problem's solution and one sentence served as the question sentence. The number of sentences, however, gradually increased across the training sessions. The number of sentences were as follows: Lessons 1 through 7 focused on identifying critical information for word problems four sentences long with one irrelevant sentence, lessons 8 and 9 focused on five-sentence-long word problems with two irrelevant sentences, lessons 10 through 15 focused on six-sentence-long word problems with three irrelevant sentences, lessons 16 and 17 focused on seven-sentence-long word problems with four irrelevant sentences, and lesson 18 through 20 focused on eight-sentence-long word problems with five irrelevant sentences.

#### Treatment fidelity

Independent evaluations were carried out to determine the treatment fidelity. During the lesson sessions, tutors were randomly evaluated by an independent observer (a post-doctoral student, a non-tutoring graduate student, and/or the project director). The observers independently filled out evaluation forms covering all segments of the lesson intervention. Points were recorded on the accuracy to which the tutor implemented the instructional sequence based off of a rubric. Observations of each tutor occurred for six sessions and was randomly distributed across instructional sessions. Inter-rater agreement was calculated on all observations and exceeded 90% across all observed categories.

### Tasks and materials

Prior to treatment implementation, a battery of group and individually administered tasks were administered. The tasks are described in detail elsewhere (Swanson et al., 2013a), but summarized below. Experimental tasks are described in more detail than published and standardized tasks. Tasks were divided into classification, pretest-only (moderator measures), and pretest/posttest measures. The sample reliabilities for each measure are reported in Table 1 and varied from 0.60 to 0.98.

### Classification measures

#### Word problems

Two measures were administered to assess word problem solving ability. The word problem subtests from the Test of Math Ability (TOMA-2; Brown et al., 1994) and KeyMath (KEYM, Connolly, 1998) were administered. Subtests from these measures yielded a scale score (*M* = 10, *SD* = 3).

#### Arithmetic computation

The arithmetic subtests from the *Wide Range Achievement Test* (WRAT-III; Wilkinson, 1993) and the *Wechsler Individual Achievement test* (WIAT; Psychological Corporation, 1992) were administered. Both subtests required written computation to problems that increased in difficulty. Problems began with simple calculations (2 + 2 =) to algebra. The dependent measure was the number of problems correct, which yielded a standard score (*M* = 100, *SD* = 15).

#### Fluid intelligence

To determine if all children were in the normal range on a measure of fluid intelligence, the *Raven Colored Progressive Matrices* (Raven, 1976, RCMT) was administered. Children were required to circle the replacement piece that best completed the patterns. After the introduction of the first matrix, children completed their booklets at their own pace. Patterns progressively increased in difficulty. The dependent measure (raw score range 0–36) was the number of problems solved correctly, which yielded a standardized score (*M* = 100, *SD* = 15).

### Working-memory (WM) measures

Three tasks were administered in this study to identify individual differences in WMC at pretest. A composite score was computed based on the z-scores of each these three tasks described below. Based on the median score z-score for the tasks below, the sample was divided into high and low WMC groups.

#### Conceptual span task

The purpose of this task was to assess the participant's ability to organize sequences of words into abstract categories (Swanson, 1992, 2013). The participant was presented with a set of words (one every 2 s), asked a discrimination question, and then asked to recall the words that “go together.” For example, a set might have included the following words: “shirt, saw, pants, hammer, shoes, nails.” The discrimination question was, “Which word, ‘saw’ or ‘level,’ was said in the list of words?” Thus, the task required participants to transform information encoded serially into categories during the retrieval phase. The difficulty of the sets ranged between two categories of two words to five categories of four words. The dependent measure was the highest set recalled correctly (range of 0–8) in which the process question was answered correctly.

#### Digit/sentence span

This task assessed the child's ability to remember numerical information embedded in a short sentence (Swanson, 1992, 2013). Before stimulus presentation, the child was shown a card depicting four strategies for encoding numerical information to be recalled. The pictures portrayed the strategies of rehearsal, chunking, association, and elaboration. The experimenter described each strategy to the child before the administration of targeted items. After all strategies have been explained, the child was then presented with numbers in a sentence context. For example, item 3 stated, “Now suppose somebody wanted to have you take them to the supermarket at 8 6 5 1 Elm Street?” The numbers were presented at 2-s intervals, followed by a process question (i.e., “What was the name of the street?”). Then, the child was asked to select a strategy from an array of four strategies that represented the best approximation of how he or she planned to practice the information for recall. Finally, the examiner prompted the child to recall the numbers from the sentence in order. No further information about the strategies was provided. Students were allowed 30 s to remember the information. Recall difficulty for this task ranged from 3 to 14 digits; the dependent measure was the highest set correctly recalled (range = 0–9) in which the process question was answered correctly.

#### Updating

Because WM tasks were assumed to tap into a measure of controlled attention referred to as updating (e.g., Miyake et al., 2000), an experimental updating task, adapted from Morris and Jones (1990), was also administered. A series of one digit numbers were presented that varied in set lengths of nine, seven, five, and three. No digit appeared twice in the same set. The examiner told the child that the length of each list of numbers might be three, five, seven, or nine digits. Participants were then told that they should only recall the last three numbers presented. Each digit was presented at approximately 1-s intervals. After the last digit was presented, the participant was asked to name the last three digits in order. In contrast to the aforementioned WM measures that involved a dual-task situation where participants answered questions about the task while retaining information (words or spatial location of dots), the current task involved the active manipulation of information such that the order of new information added to or replaced the order of old information. That is, to recall the last three digits in an unknown (*N* = 3, 5, 7, 9) series of digits, the order of old information must be kept available (previously presented digits), along with the order of newly presented digits. Thus, task performance reflected the activity of both the phonological system as well as the executive system. The dependent measure was the total number of sets correctly repeated (range 0–16).

### Pretest and posttest measures

#### Targeted measure of word problem solving accuracy

Because children were classified as at risk for MD on the TOMA and KeyMath, a separate norm-referenced measure of word problem solving accuracy was administered at pretest and posttest: the Story Problem subtest from the Comprehensive Mathematical Abilities Test (CMAT; Hresko et al., 2003). The technical manual for this subtest reported adequate reliabilities (>0.86) and moderate correlations (>0.50) with other math standardized tests (e.g., the Stanford Diagnostic Mathematics Test). The test included story problems that increased in solution difficulty. Two forms of the measures were created that varied only in names and numbers. The two forms were counterbalanced across presentation order.

### Transfer measures

We were interested in how well treatment effects that combined strategy instruction with a practice that included a gradual increase in identifying irrelevant proposition would generalize to working memory tasks. Two working memory tasks were administered.

#### Operation span

A version of the Turley-Ames and Whitfield (2003) operation span task, modified for children (Swanson et al., 2010), was administered at pretest and posttest. Two identical forms were created and counterbalanced for presentation order. The operation span test assessed WM span by having participants solve simple math problems while remembering unrelated to-be-remembered (TBR) words that followed each math problem. After each simple addition or subtraction operation, a TBR word was visually and orally presented for later recall. Our measure differed from those in the Turley-Ames and Whitfield tasks in two ways. First, a list of high-frequency words derived from Fry's Most Frequently Used Word List and the Dolch reading list served as the TBR words for pre- and post-operation span measures. Second, only one-digit addition and subtraction math problems were used. Prior to the study, TBR words were assigned randomly to math operations. Similar to Turley-Ames and Whitfield measures, operation-word sequences were presented in five parts: (a) a number from 1 to 18, (b) an addition or subtraction sign, (c) a number from 1 to 18, and (d) “ = ____.” When the “d” part of the operation was presented, the participant read the math problem aloud, reported an answer, and the experimenter recorded the participant's answer. After providing an answer for the math problem, the TBR word was revealed for 5 s and read aloud by the participant.

Operation-word sequences were presented in increasing set size. Children completed two practice trials with a set size of two. Children were then presented with operation-word sequences in sets of 2, 3, 4, and 5 with two trials for each set size for a total of 10 sets. Children received points toward their span score for correctly solving the math problems, for the number of correctly recalled words, and for the correct order of word recall. This scoring procedure was implemented to prevent giving participants credit for recalling words at the expense of solving the math problems incorrectly.

#### Visual matrix task

The purpose of this task was to assess the ability of participants to remember visual sequences within a matrix (Swanson, 1992, 2013). Participants were presented a series of dots in a matrix and were allowed 5 s to study the matrix. The matrix was then removed and participants were asked, “Are there any dots in the first column?” To ensure the understanding of columns prior to the test, participants were shown the first column location and practiced finding it on blank matrices. In addition, for each test item, the experimenter pointed to the first column on a blank matrix (a grid with no dots) as a reminder of the first column location. After answering the discriminating question (by circling “Y” for yes or “N” for no), students were asked to draw the dots they remembered seeing in the corresponding boxes of their blank matrix response booklets. The task difficulty ranged from a matrix of four squares and two dots to a matrix of 45 squares and 12 dots. The dependent measure was the highest set recalled correctly (range of 0–11) in which the process question was answered correctly.

### Covariate

Several studies have found that WM was unrelated to problem solving accuracy when reading proficiency scores were entered into the regression analyses (Swanson et al., 1993; Fuchs et al., 2006). Thus, it was necessary to administer reading measures at pretest because of their potential to partial out the effects of WM on problem solving accuracy in post-test treatment outcomes.

#### Word recognition

Word Recognition was assessed by the reading subtest of the WRAT-III. The task provided a list of words of increasing difficulty. The child's task was to read the words until 10 errors occurred. The dependent measure was the number of words read correctly.

#### Reading comprehension

Reading comprehension was assessed by the Passage Comprehension subtest from the Test of Reading Comprehension (TORC-III, Brown et al., 1995). The purpose of this task was to assess the child's comprehension of topic or subject meaning's during reading activities. Comprehension questions were drawn from the reading of short-paragraphs. The dependent measure was the number of questions answered correctly.

## Results

Table 1 provides the means, standard deviations, and reliability (Cronbach α) of the measures for the total sample. The means and standard deviations were further divided into children with and without MD, and further divided into high and low working memory span groups based on a median split of the WM composite score (mean z-score of updating, digit-sentence span, conceptual span) administered at pretest. As expected from a median split of the total sample, children with MD were more likely to yield low WM span scores (67% of MD sample) than children without MD (40%), χ^2^_(1, *N* = 204)_ = 13.87, *p* < 0.001. Thus, it is important to note in our sample that not all children with MD in problem solving suffered from low WM skills.

For analyses purposes, post-test criterion measures were converted to z-scores based on pretest performance (*M* = 0, *SD* = 1). The z-score transformation allowed for comparison across various dependent measures as well as the identification of outliers (absolute z-score > 3.5). There were no outliers e identified in this data set. Table 2 provides the posttest z-scores based on the mean and standard deviations at pretest, as well as posttest scores adjusted for pretest and the reading composite scores. Also reported are the gain z-scores (posttest minus pretest) that were uncorrected for pretest performance.

**Table 2:** **Z-scores for posttest, gain, and adjusted posttest scores as a function of treatment conditions, md status, working memory level, and criterion measures**.

**Variable**	**Verbal-emphasis**			**Verbal + Visual**			**Visual-emphasis**			**Control**		
	**Mean**	***SD***	**ADJ**	**Mean**	***SD***	**ADJ**	**Mean**	***SD***	**ADJ**	**Mean**	***SD***	**ADJ**
**MD-LWM**												
CMAT2^a^	−0.37	0.80	0.28	0.03	1.06	0.42	−0.45	1.37	0.10	−0.32	0.87	0.21
Visual-Span2	0.06	1.01	0.007	0.63	1.09	0.52	−0.13	0.94	−0.05	−0.51	0.79	−0.36
Oper-Span2	0.03	1.02	0.40	0.17	0.74	0.21	0.21	0.76	0.29	0.14	0.76	0.40
*Gain Scores*												
CMAT-G	0.59	0.43		0.54	0.69		0.28	0.58		0.29	0.82	
Visual-Span-G	0.27	1.3		0.85	1.08		0.52	1.00		−0.26	1.1	
Oper-Span-G	0.49	0.66		0.15	0.33		0.25	0.33		0.37	0.54	
**MD-HWM**												
CMAT2^a^	0.70	0.43	0.52	0.06	1.09	0.15	0.55	0.56	0.43	0.24	0.93	0.08
Visual-Span2	0.10	1.22	−0.12	0.34	0.98	0.21	1.65	0.53	1.54	−0.74	0.47	−0.39
Oper-Span2	0.24	0.93	0.32	0.25	0.8	0.22	1.42	1.00	1.75	0.5	1.25	0.29
*Gain Scores*												
CMAT-G	0.72	0.85		0.30	0.38		0.33	0.8		0.07	0.27	
Visual-Span-G	−0.79	1.43		−0.22	1.33		1.38	1.36		−0.24	1.05	
Oper-Span-G	0.42	0.68		0.23	0.3		1.87	1.25		0.19	0.48	
**NMD-LWM**												
CMAT2^a^	0.82	0.53	0.69	0.42	0.53	0.19	0.47	0.71	0.42	0.74	0.57	0.75
Visual-Span2	0.06	1.09	−0.02	0.83	1.37	0.93	−0.03	0.77	0.02	0.04	0.7	0.14
Oper-Span2	−0.04	0.64	0.13	0.04	0.75	0.25	0.80	1.05	0.96	0.59	0.96	0.43
*Gain Scores*												
CMAT-G	0.65	0.64		0.11	0.27		0.42	0.74		0.74	0.77	
Visual-Span-G	−0.2	0.97		1.19	1.73		0.09	1.17		0.35	1.13	
Oper-Span-G	0.21	0.56		0.35	0.55		0.91	1.02		0.35	0.76	
**NMD-HWM**												
CMAT2^a^	1.20	0.6	0.58	0.96	0.89	0.52	0.94	0.53	0.49	0.42	0.87	0.23
Visual-Span2	0.64	0.94	0.59	0.93	0.84	0.87	0.08	0.85	0.05	−0.03	0.93	−0.23
Oper-Span2	0.79	1.12	0.45	0.83	1.33	0.41	0.45	0.72	0.42	0.24	1.01	0.39
*Gain Scores*												
CMAT-G	0.41	0.39		0.46	0.61		0.41	0.63		0.16	0.49	
Visual-Span-G	0.36	1.18		0.8	1		0.16	1.03		−0.48	1.24	
Oper-Span-G	0.39	0.78		0.32	0.64		0.48	0.7		0.48	0.71	

ADJ, Adjusted mean post-test for covariates and cell size; LWM, low working memory; HWM, high working memory, Verbal-emphasis, N = 11 for MD-LWM and N = 10-MD-HWM, N = 12 for non NMD-LWM and N = 16 for NMD-HWM, Verbal + Visual Strategies, N = 20 for MD-LWM and N = 12 MD-HWM, N = 6 for non MD-LWM and N = 17 for NMD-HWM, Visual-emphasis, N = 15 for MD-LWM and N = 4 MD-HWM, N = 10 for non MD-LWM and N = 15 for NMD-HWM. Control, N = 20 for MD-LWM and N = 5 MD-HWM, N = 15 for NMD-LWM and N = 14 for NMD-HWM.

a Two after measure is the posttest score, _g, gain score; CMAT, Comprehensive Math Abilities Test; Oper, Operation Span; Visual-span, Visual matrix span measure.

For archival purposes, Appendix A in Supplementary Materials shows the raw pretest, posttest, and gain performance as a function of treatment conditions (Verbal-emphasis, Verbal + Visual Strategy, Visual-emphasis, and control), MD status (non MD vs. MD), and WM span (high vs. low), respectively. Also reported are the sample sizes for each treatment as a function of the subgroups.

### Comparisons at pretest

Prior to analyzing treatment effects at post-test, comparison was made between pretest measures as a function of treatment conditions as well as a function of math and WMC subgroups.

The criterion measures used to assess treatment effects were the CMAT, Operation Span, and Visual Matrix Span. A MANCOVA was computed between the four treatment conditions at pretest on these criterion measures. The MANOVA was not significant, Wilks' Λ = 0.94, *F*_(9, 464)_ = 1.39, *p*>0.05. One-Way ANOVAs were also computed on fluid intelligence (Raven), reading composite scores (WRAT-III and TORC), and the WM composite score as a function of treatment conditions. The results were non-significant for the fluid intelligence,*F*_(3, 198)_ = 1.22, *p*> 0.05, reading, *F*_(3, 198)_ = 1.54, *p*>0.05, and for the WM composite score, *F*_(3, 198)_ = 0.51, *p*>0.05.

Although children were randomly assigned to treatment conditions, it was necessary to determine if preexisting differences emerged on demographic and classification measures. A chi-square test indicated no significant differences emerged among the 4 treatment conditions as a function of MD status, χ^2^_(3, *N* = 204)_ = 2.15 *p* > 0.05, or gender, χ^2^_(3, *N* = 204)_ = 4.88, *p* > 0.10. In addition, no significant differences emerged in the proportion of high and low WM span groups across treatment conditions, χ^2^_(3, *N* = 204)_ = 2.83, *p* > 0.05. A further comparison was made amongst the classification measures between the two math groups. A MANOVA was computed between children with MD and without MD (NMD) on standard scores for problem solving (TOMA, Key Math, CMAT), reading (WRMT, WRAT), RCMT, and math calculation (WRAT, WIAT). As expected, the MANOVA was significant, Wilks'Λ = 0.27, *F*_(6, 178)_ = 78.67, *p* < 0.001. All the univariates (*p*s < 0.05) were significant and in favor of children without MD. The standard scores are shown in Table 1. It is important to note that although fluid intelligence, reading, and calculation scores were in the normal range for children with MD, children without MD had a clear advantage across these aptitude and achievement measures.

### Post-test performance

The primary analysis for this study was a mixed ANCOVA on post-test scores. The random effects included children nested within classrooms. In contrast to a traditional ANCOVA, where significance is tested against the residual error, the test of fixed effects in mixed models is tested against the appropriate error terms as determined by the model specification. The method also overcomes some of the limitations of a traditional ANCOVA because it does not require that missing data be ignored and provides a valid means to addressing standard errors. The estimates for criterion were based with full-information maximum-likelihood, and utilized robust standard errors (Huber-White) to allow for the non-independence of observations from children nested within the classroom. Because the cells were unbalanced and missing data, a Kenward-Roger correction was used to obtain the degrees of freedom.

#### Problem solving accuracy

A 2 (MD status: MD vs. NMD risk) × 2 (WMC: high and low WM ability) × 4 (treatment condition) mixed ANCOVA (pretest and reading as covariates) was computed on the CMAT scores. The covariate for reading was a composite score (WRAT, TORC). The results indicated a significant main effect for MD status, *F*_(1, 163)_ = 7.43, *p* < 0.01 and treatment, *F*_(3, 163)_ = 3.13, *p* < 0.01. A significant effect also occurred for the WMC x treatment interaction, *F*_(3, 163)_ = 3.56, *p* < 0.05, and the MD status × WMC × treatment interaction, *F*_(3, 163)_ = 2.65, *p* < 0.001. The covariates were significant for pretest,*F*_(1, 163)_ = 86.63, *p* < 0.001 and reading, *F*_(1, 163)_ = 19.60, *p* < 0.0001. As expected, the adjusted posttest scores were significantly lower for children with MD when compared to children without MD (Adjusted *M* = 0.28, *SE* = 0.04 vs. 0.48, *SE* = 0.04) and post-test scores were significantly (*p*s < 0.05) higher for the verbal emphasis condition when compared to other conditions (adjust *M*'s = 0.54, 0.32, 0.36, 0.32 for verbal, verbal + visual, visual emphasis and control condition, respectively).

A test of simple effects on adjusted posttest scores within treatment conditions yielded significant performance differences among subgroups the included children with MD but low WM (MD-LWM), children with MD but relatively high WM (MD-HWM), children without MD but low WM (NMD-LWM), and children without MD but high WM (NMD-HWM). Significant effects occurred for the verbal + visual condition, *F*_(3, 163)_ = 4.89, *p* < 0.1 and control condition *F*_(3, 163)_ = 3.80, *p*>0.05. No other significant effects occurred (all *p*s > 0.05). A Tukey test yielded significant (*p*s < 0.05) subgroup differences within the verbal + visual condition (MD-LWM = NMD-HWM > NMD-LWM = MD-LWM), and control condition (NMD-LWM > NMD-HWM = MD-LWM = MD-HWM).

When comparisons were made across treatment conditions within each subgroup, no significant treatment effects were found for the MD-LWM subgroup, *F*_(3, 163)_ = 1.11, *p*>0.05. Significant treatment effects were found for the MD-HWM subgroup *F*_(3, 163)_ = 4.69, *p* < 0.01 (verbal = visual > verbal + visual = control), NMD-LWM subgroup, *F*_(3, 163)_ = 10.48, *p* < 0.01 (control = verbal > visual > verbal + visual), and the NMD-HWM subgroup, *F*_(3, 163)_ = 2.97, *p* < 0.05 (verbal = verbal + visual = visual > control).

In general, the important pattern related to the three-way interaction was that children with low WMC and at risk for MD did not benefit from the strategy conditions when compared to the control conditions. Thus, we did not find support for the assumption that strategy conditions were more likely to help children with MD but low WMC, than children with MD but relatively higher WMC.

### Transfer

As before, a mixed level 2 (high vs. low risk for MD) × 2 (high and low WM ability) × 4 (treatment condition) ANCOVA (pretest and reading as covariates) was computed on posttest scores for the transfer measures.

#### Visual matrix

A mixed 2 (MD vs. NMD risk) × 2 (high and low WMC ability) × 4 (treatment condition) ANCOVA (pretest and reading as covariates) was computed on the adjusted visual-matrix scores. The results indicated a significant main effect for treatment, *F*_(3, 161)_ = 5.67, *p* < 0.01, and for the MD status x treatment interaction, *F*_(3, 161)_ = 20.47, *p* < 0.01, WMC × treatment interaction, *F*_(3, 161)_ = 2.86, *p* < 0.05, and the MD status × WMC × treatment interaction, *F*_(3, 161)_ = 3.73, *p* < 0.001. The covariates were significant for pretest, *F*_(1, 161)_ = 32.64, *p* < 0.001, but not reading, *F*_(1, 161)_ = 0.11, *p*>0.05. As expected, the adjusted posttest scores were significantly higher for children with higher WMC than lower WMC (Adjusted *M* = 0.31, *SE* = 0.07 vs.0. 15, *SE* = 0.12), and scores were significantly (*p*s < 0.05) higher for the verbal + visual condition when compared to other treatment conditions (adjust *M*'s = 0.12, 0.63, 0.39, −0.21 for verbal, verbal + visual, visual emphasis, and control conditions, respectively).

Within treatment conditions, a significant subgroup effect occurred for the verbal, *F*_(3, 161)_ = 3.04, *p* < 0.05, verbal + visual, *F*_(3, 161)_ = 17.67, *p* < 0.001, and control conditions *F*_(3, 161)_ = 3.83, *p* < 0.01. No other significant effects occurred. A Tukey test indicated that the significant (*p*s < 0.05) subgroup differences occurred within the verbal (NMD-HWM > NMD-LWM = MD-LWM > MD-HWM), verbal + visual (NMD-HWM = NMD = LWM > MD-LWM = MD-HWM) and control conditions (NMD-LWM > NMD-HWM = MD-LWM = MD-HWM).

When comparisons were made across treatment conditions within each subgroup, no significant treatment effects were found for the NMD-HWM subgroup, *F*_(3, 161)_ = 2.17, *p*>0.05, or the MD-HWM subgroup, *F*_(3, 161)_ = 1.79, *p*>0.05. Significant treatment effects were found for the MD-LWM subgroup *F*_(3, 161)_ = 2.69, *p* < 0.05 (verbal + visual > visual > verbal > control), and the NMD-LWM subgroup, *F*_(3, 161)_ = 15.90, *p* < 0.01 (verbal + visual > control > verbal = visual).

In summary, the results contrast with the post-test problem solving findings for children with MD but low WMC. The previous results suggested that the verbal + visual condition yielded significantly higher post-test visual-spatial WM scores for children with and without MD who also have low WMC when compared to other conditions.

#### Operation span

A 2 (MD vs. NMD risk) × 2 (high and low WM ability) × 4 (treatment condition) mixed ANCOVA (pretest and reading as covariates) was computed on the post-test operation span scores. The results yielded a significant effect for treatment, *F*_(3, 170)_ = 9.44, *p* < 0.01, WMC, *F*_(1, 170)_ = 4.10, *p* < 0.01, and the MD status × WMC × treatment interaction, *F*_(3, 163)_ = 2.65, *p* < 0.001. The covariates were significant for pretest, *F*_(1, 170)_ = 272.77, *p* < 0.001, but not reading, *F*_(1, 170)_ = 3.40, *p* = 0.07. The adjusted posttest scores were significantly higher for children with higher WMC when compared to children with lower WMC (Adjusted *M* = 0.53, *SE* = 0.06 vs. 0.37, *SE* = 0.03), and scores were higher for the visual emphasis condition when compared to other conditions (adjust *M*'s = 0.32, 0.28, 0.83, 0.38 for verbal, verbal + visual, visual emphasis and control condition, respectively).

Within treatment conditions, a test of simple effects on adjusted posttest scores yielded significant performance differences among subgroups within the visual emphasis condition, *F*_(3, 170)_ = 20.80, *p* < 0.01. No other subgroup differences occurred within treatments (*ps*>0.05). A Tukey test showed that significant (*p*s < 0.05) subgroup effects within the visual-emphasis condition were related to higher post-test performance for children MD and high WMC (MD-HWM > NMD-LWM > NMD-HWM > MD-LWM).

When comparisons were made across treatment conditions, no significant treatment effects were found for the MD-LWM subgroup, *F*_(3, 170)_ = 1.10, *p*>0.05, or the NMD-HWM subgroup, *F*_(3, 170)_ = 0.03, *p*>0.05. Significant treatment effects were found for the MD-HWM subgroup, *F*_(3, 170)_ = 5.46, *p* < 0.01 (visual > verbal + visual = control > verbal emphasis) and the NMD-LWM subgroup, *F*_(3, 170)_ = 4.60, *p* < 0.01(visual > control > verbal + visual > verbal).

In summary, the results indicated an advantage at post-test for the visual emphasis condition relative to the control condition for the operation span measures, but these effects were isolated to children with MD with relatively higher WMC.

### Effect sizes

In summary, a number of significant interactions for posttest outcomes occurred as a function of treatment conditions and subgroups. However, because of small sample sizes (see Appendix A in Supplementary Materials), the experiment may have been underpowered. To partially address this issue, effect sizes (ESs) were computed. We calculated Hedge's *g* = γ / [(*SD*^2^_1_) (*N*_1_) + (*SD*^2^_2_) (*N*_2_)/2]^1∕2^ where γ was the HLM coefficient for the adjusted posttest mean difference between treatment (adjusted for pretest and reading and adjusted for both level-1 and level-2 covariates), and *N*_1_ and *N*_2_ were the sample sizes. *SD*_1_ and *SD*_2_ were the standard deviations for the unadjusted posttest treatment conditions, respectively.

Table 3 shows ESs comparing each treatment within each subgroup. For the interpretation of the magnitude of the effect sizes, Cohen's (1988) distinction was used: (1) an ES of 0.20 is considered small, and (2) an ES of 0.50 and 0.80 is considered moderate and large, respectively. For the purposes of this study, only ESs above 0.50 were considered meaningful. As shown in Table 3, the first left three columns show ESs for the control condition (treatment = 4) when compared to verbal-emphasis (treatment = 1), verbal + visual (treatment = 2), and visual-emphasis (treatment = 3) conditions. A negative effect size favored the strategy conditions over the control condition.

**Table 3:** **Effect sizes on post-test means adjusted for pretest, reading and random effects**.

	**1 vs. 4**	**2 vs. 4**	**3 vs. 4**	**1 vs. 3**	**2 vs. 3**	**1 vs. 2**
**MD-LWM**						
CMAT	0.08	0.22	−0.10	0.15	0.27	−0.14
Visual-Span	0.42	**0.92**	0.36	0.06	0.55	−0.48
Oper-Span	0.01	−0.25	−0.14	0.13	−0.11	0.22
**MD-HWM**						
CMAT	**0.70**	0.07	0.44	0.19	−0.28	0.43
Visual-Span	0.26	**0.69**	**3.89**	−1.52	−1.47	−0.30
Oper-Span	0.03	−0.07	**1.27**	−1.51	−1.81	0.12
**NMD-LWM**						
CMAT	−0.11	−1.00	−0.53	0.44	−0.35	0.94
Visual-Span	−0.18	**0.85**	−0.16	−0.04	0.89	−0.80
Oper-Span	−0.36	−0.20	**0.53**	−0.98	−0.74	−0.18
**NMD-HWM**						
CMAT	0.47	0.33	0.36	0.16	0.04	0.08
Visual-Span	**0.88**	**1.25**	0.31	0.60	0.97	−0.31
Oper-Span	0.06	0.02	0.03	0.03	−0.01	0.03

1, Verbal-emphasis Condition; 2, Verbal + Visual Condition; 3, Visual-emphasis Condition, and 4, Control. Bold positive ES reflects moderate (>0.50) to high ESs (>0.80) outcomes in favor of strategy conditions relative to control condition. CMAT, Comprehensive Math Abilities Test, Oper, Operation Span, Visual-span, Visual matrix span measure.

#### Children with MD

For the MD- low WMC subgroup (MD-LWM), no meaningful effect sizes emerged related to problem solving accuracy. The only ESs of importance was the large ESs (*ES* = 0.92) in favor of the combined verbal + visual conditions relative to control conditions on post-test measures of visual-spatial WM.

For children with MD, but high WM spans, a high ES (*ES* = 0.70) occurred in favor of the verbal-emphasis treatment when compared to the control condition on the problem solving measure. A clear advantage relative to the condition was also found for the visual-emphasis condition for the visual-spatial WM transfer task (*ES* = 3.89), and the operation span transfer task (*ES* = 1.27).

#### Children without MD

For children without MD but low WM spans, no clear advantage was found for a specific strategy condition when compared to the control condition on posttest problem solving accuracy scores. An advantage at post-test was found relative to the control condition for the verbal + visual condition on the transfer measures of visual-spatial WM (*ES* = 0.85), and the visual emphasis condition for the operation span transfer measure (*ES* = 0.53).

For children without MD but high WM spans, a slight advantage was found for the verbal emphasis condition when compared to the control condition on measures of post-test problem solving (*ES* = 0.47). In addition, the verbal and verbal + visual conditions exceeded the control condition on posttest measures of visual-spatial WM (*ES* = 0.88, 1.25), whereas no strategy advantage was found for strategy conditions on the operation span measure (ES vary from 0.02 to 0.06).

## Discussion

This study investigated the role of strategy instruction on word problem solving accuracy in children with MD. Three important findings occurred. First, support was found for the notion that strategy instruction facilitates solution accuracy but the effects of strategy instruction were moderated by individual differences in WM span. Second, some strategies yielded higher post-test scores than others, but these findings were qualified as to whether children were or were not at risk for MD. Finally support was found for strategy training on problem solving measures in facilitating a transfer to working memory measures. Given these general findings, the results will now be placed within the three questions that directed this study.

### Do cognitive strategies place different demands on WMC in children with MD?

Initially, we assumed that strategy training would be more beneficial for children with MD than for children without MD. That is, we assumed that any potential three-way interactions (ability group × WMC × treatment) would reflect variations within the group of children with MD. This assumption was based on several investigations showing that children with MD are more likely to experience greater processing constraints in cognition, especially on WM tasks, when compared to children without MD (e.g., Koonz and Berch, 1996; Swanson and Beebe-Frankenberger, 2004; Andersson and Lyxell, 2007). For example, students with MD struggle on both letter and number-based WM span tasks (Koonz and Berch, 1996; see Bull and Espy, 2006, for review). Several studies also suggest that children with MD have difficulty inhibiting irrelevant information from entering WM (Bull et al., 2008). In addition, studies have shown that strategy training helps low span participants allocate WM resources more efficiently when compared to high span participants (e.g., Turley-Ames and Whitfield, 2003). Thus, we expected that children with MD, especially those with low WM span, would benefit more from strategy instruction than children without MD (children with high spans). The present results did not support this hypothesis.

The general pattern was that regardless of MD status, children with higher WM spans were more likely to benefit from strategy conditions than children with low spans. When compared to the control condition, post-test solution accuracy for children with MD but with higher WMC, yielded effect sizes within the moderate range when strategy conditions included a verbal or visual emphasis (*ES* = 0.70 and 0.44, respectively). Likewise, children without MD but with higher WMC, yielded a moderate effect size (*ES* = 0.47) related to adjusted post-test solution accuracy when strategy conditions included a verbal emphasis. In contrast, effect sizes related to post-test problem solving for strategy conditions when compared to control conditions, were in the low range for children with low WMC. Thus, there is weak support for the assumption that strategy training is more advantageous for children with low WMC than high WMC on post-test measures of problem solving.

### Are some cognitive strategies more effective than others for children with MD?

The results were clear in answering this question. No strategies that included low span children with MD yielded post-test effect sizes in the moderate range. In contrast, high span children with MD were more likely to yield post-test effect sizes in the moderate to high range for the verbal or visual-emphasis strategy conditions. The results do present a different picture, however, when post-test measures included visual-spatial WM. A post-test advantage was found for children with MD and low WMC when strategy conditions combined verbal and visual information (verbal + visual condition, *ES* = 0.92). Likewise, children with MD but with high WMC improved in visual-spatial WM when conditions included visual information (verbal + visual, and visual emphasis, *ES* = 0.69 and 3.89, respectively). Based on the assumption that visual WM in children with MD is relatively intact (Swanson and Jerman, 2006), we anticipated that visual-spatial strategies would yield higher accuracy scores when compared to verbal strategy conditions. The results showed that both high and low WM span groups benefitted from visual strategies, however children with low WM span needed the combination of both verbal and visual strategies.

### Does practice solving problems that gradually increase irrelevant information influence WM performance?

We found partial support for the assumption that problem solving training facilitated improvement in WM performance. We assumed this occurred because word problem solving required focused attention to relevant propositions in text in the face of irrelevant propositions; and strategy training helped children focus attention to relevant propositions, which in turn, influenced solution accuracy. Likewise, we assumed that practice in controlled-attention, i.e., activities that maintain (e.g., update) information in the face of interference or distraction, influenced WM performance (see Engle et al., 1999; Kane and Engle, 2003, for a review). We say “partial support” for this finding because only children with MD and relatively high WMC capacity improved on both transfer measures (visual-span and operation span) as a function of the same instructional condition (visual-emphasis treatment). The only other group to show transfer to both WM measures included children without MD but low WM. We have no explanation for this finding. Part of the difficulty of unraveling this interaction is that practice related to solving problems with increasing interference (gradual increases in irrelevant sentence proposition) was not separated from the overt cognitive strategy instruction. Thus, we cannot infer that such practice enhanced transfer to the WM measures.

The results do inform current controversies, however, on the influence of WM training on academic performance. For example, in an analysis by Kane et al. (2007) on WM strategy training studies, they concluded that although strategies may improve WM performance, the post-test outcomes reveal a weak relationship between WM span and achievement. Our results suggest, however, that academic tasks that training processes related to WM (controlled attention) may in fact influence later WM performance. This inference on our part is consistent with several studies that suggest WM is related to attentional control (e.g., Engle et al., 1999; Bayliss et al., 2003; Kane et al., 2007), and attentional control is important when performing complex problem solving tasks (e.g., Kyllonen and Christal, 1990; Unsworth, 2010).

### Limitations

There are at least two limitations to this study. The first is that sample size was small for some of the cells. This was especially true when identifying high WM span participants in the sample with MD and the low WM span participants in the sample of children without MD. Thus, there may be a loss of power in testing for significant interactions. The magnitude of the effect sizes does show, however, that high span participants with MD status benefited from the strategy conditions across a number of dependent measures.

Second, the control treatment conditions were highly effective in yielding positive gains in post-test performance. The schools in which the study was implemented utilized an evidence-based math curriculum and teachers within each classroom placed a high emphasis on fluency in mathematical skills. Although we showed gains in problem solving performance for the majority of children in the strategy conditions relative to this control condition, not all children benefited from the strategy conditions. For example, strategy conditions had no significant influence on solution accuracy on CMAT for low span children without MD. We have no explanation for this finding except that perhaps the school wide curriculum is well matched to this sample.

### Implications

Our findings have two applications to current research. First, the results are consistent with studies suggesting that strategies facilitate problem solving for children with MD. However, those strategies that are most beneficial must be adapted to the WM level of the child. A second application relates to interventions to designed to improve WM. No studies we are aware of have shown that WM training directly influences academic outcomes. The alternative we took to enhance transfer, was to embed WM demands within the curriculum and to provide children with strategies to handle these increased WM demands. Although the mechanism that underlies this transfer is unclear, we did find transfer in two groups of children: (1) those with high WMC, but low achievement, and (2) those with low WMC but high achievement. Thus, further studies that place WM demands within the curriculum would potentially clarify those mechanisms.

In summary, the results suggest that WMC moderates treatment outcomes for children MD. Unfortunately, these outcomes are primarily isolated across the majority of measures to children with relatively higher WMC.

### Conflict of interest statement

The author declares that the research was conducted in the absence of any commercial or financial relationships that could be construed as a potential conflict of interest.
