# Supplementary material for: Cognitive strategy interventions improve word problem solving and working memory in children with math disabilities
Source: Front Psychol. 2015 Aug 4;6:1099. doi: 10.3389/fpsyg.2015.01099 (PMC4523823; doi:10.3389/fpsyg.2015.01099)
Supplement: Supplementary file 1 [file DataSheet1.PDF]

Appendix A

Means, Standard Deviations and Gains for Raw Scores for Each Subgroup and Treatment Condition
Raw Scores

	Verbal-emphasis		Verbal + Visual		Visual-emphasis		Control	
Variable	Mean	SD	Mean	SD	Mean	SD	Mean	SD
MD-LWM								
CMAT	5.09	2.34	6.50	3.27	5.80	3.3	6.60	2.98
CMAT2	6.91	2.47	8.15	3.27	6.67	4.22	7.08	2.69
CMAT-G	1.82	1.33	1.65	2.13	0.87	1.77	0.90	2.53
Vis-Span	12.00	7.00	11.63	7.77	8.63	5.84	12.95	7.59
Vis-Span2	14.27	8.57	19.05	9.25	12.6	8.01	9.42	6.73
Vis-Span-G	2.27	11.08	7.26	9.16	4.47	8.54	-2.25	9.36
Oper-Span	2.73	2.20	4.89	3.78	4.5	3.74	4.20	3.9
Oper-Span2	4.82	4.38	5.40	3.15	5.56	3.24	5.29	3.26
Oper-G	2.09	2.81	0.63	1.42	1.06	1.44	1.60	2.33
MD-HWM								
CMAT	8.00	2.87	7.33	3.03	8.75	1.71	7.67	3.27
CMAT2	10.20	1.32	8.25	3.36	9.75	1.71	8.8	2.86
CMAT-G	2.20	2.62	0.92	1.16	1.00	2.45	0.20	0.84
Vis-Span	21.3	8.77	18.50	11.65	16.00	11.83	10.33	7.42
Vis-Span2	14.6	10.37	16.67	8.33	27.75	4.5	7.40	4.04
Vis-Span-G	-6.70	12.14	-1.83	11.33	11.75	11.59	-2.00	8.97
Oper-Span	3.90	4.25	4.75	4.22	2.75	3.1	6.00	5.33
Oper-Span2	5.70	3.97	5.75	3.44	10.75	4.27	6.83	5.34
Oper-G	1.80	2.9	1.00	1.28	8.00	5.35	0.83	2.04
NMD-LWM								
CMAT	8.58	1.56	9.14	1.68	8.83	2.89	8.19	2.93
CMAT2	10.58	1.62	9.33	1.63	9.5	2.17	10.33	1.76

CMAT-G	2.00	1.95	0.33	0.82	1.3	2.26	2.27	2.37
Vis-Span	15.92	9.48	13.86	11.6	13	7.32	11.07	6.53
Vis-Span2	14.25	9.26	20.83	11.63	13.5	6.54	14.07	5.93
Vis-Span-G	-1.67	8.29	10.17	14.72	0.80	9.98	3.00	9.65
Oper-Span	3.58	3.00	3.67	4.41	4.17	4.55	5.69	4.39
Oper-Span2	4.50	2.75	4.86	3.24	8.08	4.52	7.19	4.1
Oper-G	0.92	2.39	1.50	2.35	3.92	4.36	1.50	3.25
NMD-HWM								
CMAT	10.50	2.28	9.59	2.87	9.67	2.41	9.29	2.62
CMAT2	11.75	1.84	11.00	2.74	10.93	1.62	9.36	2.68
CMAT-G	1.25	1.18	1.41	1.87	1.27	1.94	0.50	1.51
Vis-Span	16.13	9.14	14.82	8.44	13.07	7.35	15.00	7.74
Vis-Span2	19.19	8.02	21.65	7.18	14.4	7.2	13.46	7.91
Vis-Span-G	3.06	10.01	6.82	8.49	1.33	8.75	-4.08	10.58
Oper-Span	6.38	5.33	6.88	5.99	4.53	4.14	3.65	4.11
Oper-Span2	8.06	4.81	8.24	5.72	6.6	3.09	5.71	4.34
Oper-G	1.69	3.34	1.35	2.74	2.07	2.99	2.06	3.03

Note. 2 after measure is posttest, g after measure is gain score, CMAT = Comprehensive Math Abilities Test , Oper = Operation Span, Visual-span = Visual matrix span measure, WIAT = calculation on the Wechsler Achievement Test, WRAT = arithmetic subtest on the Wide Range Achievement Test, LWM = low working memory, HWM = high working memory, Verbal-emphasis, *N* = 11 for MD-LWM and *N* = 10-MD-HWM, *N* = 12 for non NMD-LWM and *N* = 16 for NMD-HWM, Verbal + Visual Strategies, *N* = 20 for MD-LWM and *N* = 12 MD-HWM, *N* = 6 for non MD-LWM and *N* = 17 for NMD-HWM, Visual-emphasis, *N* = 15 for MD-LWM and *N*=4 MD-HWM, *N* = 10 for non MD-LWM and *N* = 15 for NMD-HWM. Control, *N* = 20 for MD-LWM and *N* = 5 MD-HWM, *N* = 15 for NMD-LWM and *N* = 14 for NMD-HWM.
